# *Dinophysis* Species and Diarrhetic Shellfish Toxins: 20 Years of Monitoring Program in Andalusia, South of Spain

**DOI:** 10.3390/toxins11040189

**Published:** 2019-03-29

**Authors:** Raúl Fernández, Luz Mamán, David Jaén, Lourdes Fernández Fuentes, M. Asunción Ocaña, M. Mercedes Gordillo

**Affiliations:** Laboratory for the Quality Control of Fishery Resources, Agency for the Management of Agriculture and Fisheries of Andalusia, Ministry for Agriculture, Fisheries and Rural Development, Regional Government of Andalusia, 21459 Andalusia, Spain; luz.m.menendez@juntadeandalucia.es (L.M.); david.jaen@juntadeandalucia.es (D.J.); lourdes.fernandez.fuentes@juntadeandalucia.es (L.F.F.); mariaa.ocana@juntadeandalucia.es (M.A.O.); mariam.gordillo@juntadeandalucia.es (M.M.G.)

**Keywords:** *Dinophysis*, Diarrhetic shellfish toxins, marine biotoxins, blooms

## Abstract

In Andalusia, the official monitoring program for toxic phytoplankton and marine biotoxins was launched in 1994 to comply with European legislation. Since then, there have been numerous episodes of DST (Diarrhetic shellfish toxins) associated with the proliferation of *Dinophysis* species. This article reviews two decades of time series data and assesses the effectiveness of the program established. The testing of lipophilic toxins and toxic phytoplankton is based on official methods harmonized and accredited since 2007 according to the standard UNE-EN-ISO 17025. The major species of *Dinophysis* identified were *D. acuminata* complex, *D. caudata*, *D. acuta* and *D. fortii*, with the main growth season being from early spring until the end of autumn. Both *D. acuminata* complex and *D. acuta* have been clearly associated with toxicity in molluscs. Despite the complexity of data obtained through monitoring programs, it is possible to provide early warning of potential health risks for most situations. This is the first report of *Dinophysis* species and their relation to DST events in a time series from Andalusia.

## 1. Introduction

The implementation of monitoring programs for marine biotoxins in the wake of the findings of Yasumoto [1,2], at a scale dependent on the geographical scope of each competent administration, started in Andalusia in 1994. These findings demonstrated a relationship between outbreaks of diarrheal intoxication in humans, the ingestion of molluscs and the presence in the environment of *Dinophysis* *fortii* that was identified as the producer of toxins responsible for poisoning. This led to the monitoring programs to carry out both analysis of the mollusc toxin content and cell counts of the phytoplankton species whose toxicity was proven over time.

The European regulations applicable concerning food security [3,4] lay down the basic requirements and recommendations for the implementation of these programs. The program implemented in Andalusia has observed the guidelines established for the definition of areas and the frequency of monitoring. This study presents the data obtained from the start of monitoring and their analysis to answer the following question: Are the regulatory requirements for the analysis of toxic phytoplankton effective as an early-warning system for the risk of contamination in molluscs on the Andalusian coast?

The identification of some species of *Dinophysis* has led to much discussion and many scientific publications. The main issues have been reviewed by Reguera et al. [5]. Identification has been carried out based on morphometric characteristics mainly using light microscopy (LM), but the considerable intraspecific variability makes it difficult to clearly discriminate between some of the species. Nevertheless, LM is the method most used by monitoring programs. In any case, in Andalusia, the set of species given in the IOC Taxonomic Reference List of Toxic Microalgae [6] has been used as the basis for monitoring, and the region has acted according to the principle of maximum food security in situations of any doubt.

A group of new causative agents of shellfish poisoning, the Diarrhetic Shellfish Toxins (DST), was isolated and their structures determined in the 1980s [1,2]. In Andalusia, many cases of DST have been detected during the years of monitoring but not all the toxins in this group have been individually characterized.

Accumulation of the toxicity produced by microalgae in filter-feeders has also been the subject of study. However, important intra- and interspecific variability has been measured resulting from causes such as differences in filtration and clearance rates [7,8] or from selection mechanisms based on the quality and quantity of prey [9,10,11]. Such variability has become a parameter considered in the monitoring program.

For more than 20 years, species of the genus *Dinophysis* have been commonly found in almost all the sampling stations in Andalusia. The species clearly associated with toxicity phenomena in molluscs, have been *D. acuminata*/*D. ovum* and *D. acuta*. The accumulation of toxins above permitted levels triggers the activation of closure protocols for fisheries that can sometimes be prolonged for up to 6 months. All the species of the genus identified in the samples that at some time have formed part of the IOC list, are presented in this study along with toxicity levels of molluscs in each case when this has occurred.

## 2. Results

The distribution of areas in Andalusia (see Section 4.1) represents the complete set of mollusc habitats ranging in size between 12.6 ha and the 35340.5 ha (average area 4842.4 ha). Smaller areas correspond to the perimeter of areas where aquaculture facilities are located.

All the information presented in this study comes from the sampling stations located in the above-mentioned areas. In each sampling, the choice of sampling points has been arbitrary within each area, except in areas where physico-chemical measurements of the water were already being made, at previously fixed points. Mollusc samples were usually selected at those points where there was high fishing activity.

### 2.1. Species of the Genus Dinophysis

Twenty *Dinophysis* species out of more than 100 that make up the genus [12] were identified on the Andalusian coast. Those *Dinophysis* cells considered gametes have been excluded from the reported counts. *Dinophysis* cells not identified to species level are reported as *Dinophysis* spp. Table 1 provides a species list along with the frequency of occurrence. The number of samples collected in the study period (20 years) was 34,329.

The difficulty in discriminating between *D. acuminata* and *D. ovum* has led us to follow the example of Lassus and Bardouil [13] or Bravo [14] who introduced the term “*Dinophysis acuminata* complex” that encompasses both, reflecting its great morphological variability. 

The species with the highest frequency were *D. acuminata* complex, *D. caudata*, *D. acuta* and *D. fortii*.

Nearly all the species were detected in both the Atlantic and the Mediterranean areas, but some show marked differences in their repartition between the two areas. While *D. acuminata* complex, *D. acuta* and *D. rotundata* (*P. rotundatum)* are more frequent on the Atlantic coast, *D. fortii* and *D. rapa* (*P. rapa*) are more frequent in the Mediterranean Sea.

Taking into account that the sampling frequency was uniform all year long, it can be observed that *D. acuminata* complex is present almost all throughout the year, although it does not always trigger toxic outbreaks in molluscs (see Section 2.3).

The couple *D. acuminata* complex/*D. caudata* is frequent in the samples. In addition, it is accompanied at times by *D. acuta* and *D. fortii.* Similar associations have already been reported in temperate waters [5].

The set of species identified as *Dinophysis* spp. occurs very frequently in both sea areas. Although these species not known to be toxic, unusual high concentrations of some of them activated alert protocols.

### 2.2. Temporal Episodes of Dinophysis

The volume of data generated during the 20 years of monitoring subject to examination is very high, and data processing is needed to condense the redundancies into manageable form. 

**Figure 1:**
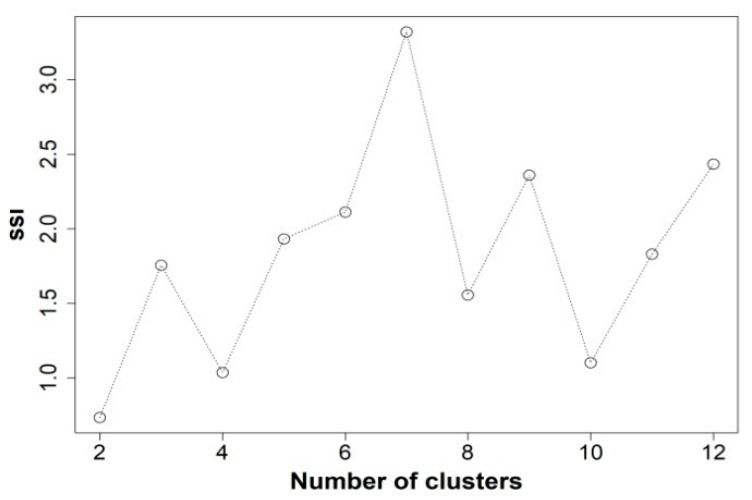
Optimizing the number of clusters through the SSI criterion. Number of samples analyzed 6659.

Figure 1 shows the choice of the appropriate number of clusters, to summarize the data, through the highest Simple Structure Index (SSI) [15]. It shows how the grouping of seven clusters best explains the variance in the data taking up 12 SSI clusters at maximum. 

Figure 2 shows the distribution of the stations in the different groups. Appendix A (Figure A1, Figure A2, Figure A3, Figure A4, Figure A5, Figure A6 and Figure A7) and Appendix B (Figure A8, Figure A9, Figure A10 and Figure A11) shows a historical series of 20 years of their abundance values, along with toxin values in molluscs of a selected area of each cluster obtained.

As discussed in the previous section, the most frequent species in the coast of Andalusia are *D. acuminata* complex, *D. caudata*, *D. acuta* and *D. fortii*. Of these, *D. acuminata* complex and *D. acuta* are the only ones which have been partnered by co-occurrence with episodes of DST. Thus, the description of episodes presented in this work will be focused on both species. 

**Figure 3:**
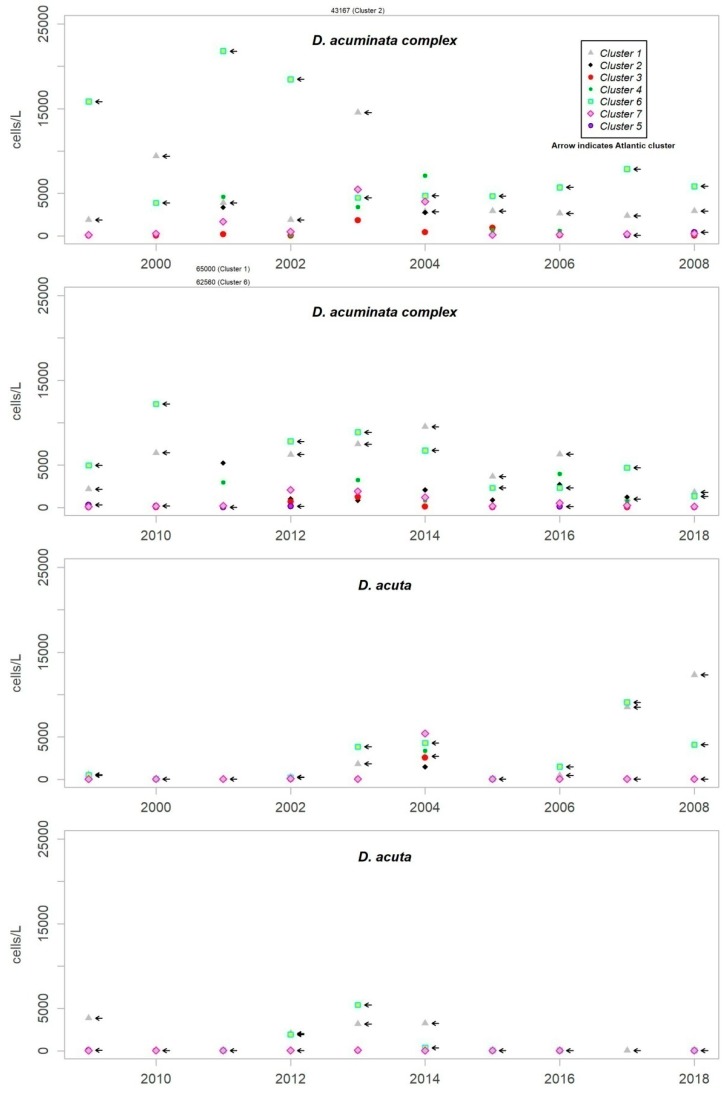
Annual maximum concentration (cells/L) of *Dinophysis acuminata* complex and *Dinophysis acuta* in each cluster throughout the monitoring period.

Although *D. acuminata* complex is present almost all year round, the more substantial episodes are usually produced as a succession of between one and three pulses from the beginning of spring until the end of autumn. The period of growth of *D. acuta* is, however, more reduced, from summer to early autumn. Although the pattern mentioned for *D. acuminata* complex has been repeated in all the years of monitoring, *D. acuta* does not present such a recurrence, and they generally disappear for one or more years. The patterns found show similarity between different years of monitoring, while the abundance reached determines the degree of difference. Figure 3 shows the maximum levels of abundance where the difference can be seen.

Although the presence of *D. acuminata* complex and *D. acuta* in the environment occurs during similar periods all along the coast of Andalusia, in general, there are notable differences in the intensity of the abundance between clusters 1 and 6 (Atlantic area) and the rest. Therefore, although there are frequent blooms of relatively high abundance in the Atlantic area, in the Mediterranean it is rare that either species exceeds 400 cells/l. However, cluster number 5 belongs to the Atlantic area and does not show the behaviour of the other neighbouring Atlantic clusters.

It sometimes happens, however, that a particular episode (characterized by a certain intensity) spreads to almost all the Andalusian coast in one direction or the other. Possible advection through the Strait of Gibraltar is suggested by the proximity of the dates of the growth pulses on both sides of the strait, but which also should be investigated using hydrodynamic models. In August 2001, August 2003, October 2004, July 2012, July 2013 and August 2014, episodes of *D. acuminata* complex were recorded with these features. *D. acuta* can be often detected along the entire coast, but not always; in August 2004, for example, a single case of unusually high intensity was recorded in all the areas monitored.

Episodes of special intensity of *D. acuta* occurred from the end of June to end of August 2007 and of *D. acuminata* complex from March to mid-April 2011, both in clusters 6 and 1. In the first episode, there were more than 2000 cells/L in 24.9% of the samples and more than 5000 cells/L in 4.7% (349 samples). A maximum value of 9080 cells/L occurred in cluster 6. In the second episode, a maximum of 65,000 cells/L (Cluster 1) was recorded, which was the highest concentration of *Dinophysis* species found during the years of monitoring. In this case, there were more than 2000 cells/L in 69.4% of the samples and more than 5000 cells/L in 47.9% (278 samples).

From 2014 on, a declining trend of abundance has been recorded for both species towards the western end (clusters 1 and 6). The *D. acuminata* complex had never shown this behavior (frequency of values above 2000 cells/L was drastically reduced) in the monitoring period.

The timing and abundance of other *Dinophysis* species is shown in Appendix B. None of them shows any obvious seasonal pattern or other type of temporary recurrence. The only condition found is spatial, so that while some species are detected in all areas sampled, others preferably appear in the Atlantic or the Mediterranean areas.

### 2.3. Toxicity in Molluscs

The criteria of the official monitoring programme aims to categorize some species of bivalve molluscs as indicators of each production area (administrative spatial unit). Therefore, in Andalusia, the species which have been monitored in areas of external waters where there have been major incidents with *Dinophysis*, have mainly been *Donax trunculus*, *Chamelea gallina*, *Mytilus galloprovincialis*, *Callista chione*, *Venus verrucosa* and *Cerastoderma edule*.

Levels of okadaic acid have been detected in all these areas when *D. acuminata* complex and/or *D. acuta* were present in the environment. There have also been cases, albeit few, in which samples showed toxicity but in which *Dinophysis* species or other organisms considered as DSP producers were not found; there were more cases in the period when the mouse bioassay method was used. Other substances that are part of the group of lipophilic toxins have been detected although on a smaller number of occasions. Levels of DTX-2 began to be measured at the beginning of June 2018 in *Donax trunculus* and *Mytilus galloprovincialis*. The episode has not finished yet. In the environment, the presence of mainly *Prorocentrum* cf. *texanum* has been accompanied by minor amounts of *D. acuminata* complex and *D. acuta*. Also, levels of yessotoxin were measured only in *Mytilus galloprovincialis* in the spring and summer of 2015 when in the water samples the presence mainly of *D. acuminata* complex was detected and, to a lesser extent, *D. acuta*, *D. fortii*, *D. caudata* and *P. rotundatum*.

**Figure 4:**
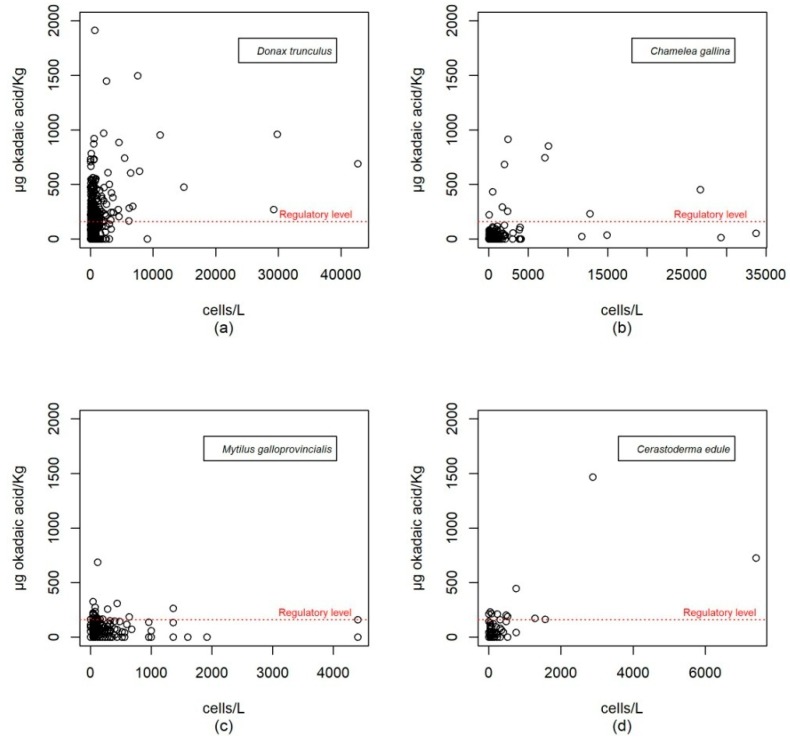
Toxicity in molluscs (µg okadaic acid/kg) versus concentration of *D. acuminata* complex + *D. acuta* (cells/L). The bivalve species with the highest toxic incidence have been selected. Data are not shown for mollusc species which, although they have a sufficient amount of data, have shown very little tendency to accumulate toxin. (**a**) *Donax trunculus;* (**b**) *Chamelea gallina;* (**c**) *Mytilus galloprovincialis;* (**d**) *Cerastoderma edule.*

The durations of the episodes usually correspond to the maintenance of the population of that or those phytoplankton species that generate them. There is thus good association between the detection of both parameters in qualitative terms. However, as it can be seen in Figure 4 which shows the pairs of cell concentration-toxicity values of parallel sampling, the levels of okadaic acid in molluscs do not correlate well with the concentration of the producer microalgae if the expected linear relationships are taken into account. Thus, it is not uncommon to find high values of okadaic acid with low values of phytoplankton and *vice versa*. 

Although there is not a good correlation between toxicity values in molluscs and the concentration of *Dinophysis* in parallel sampling, if each episode as a whole is considered, toxicity patterns in molluscs do correspond to the patterns of the producer phytoplankton species as might be expected. Thus, in general, there have been more incidents in the Atlantic area than in the Mediterranean area; episodes have occurred in warm seasons and there has been a trend toward a decrease in measured values over the last three years.

**Figure 5:**
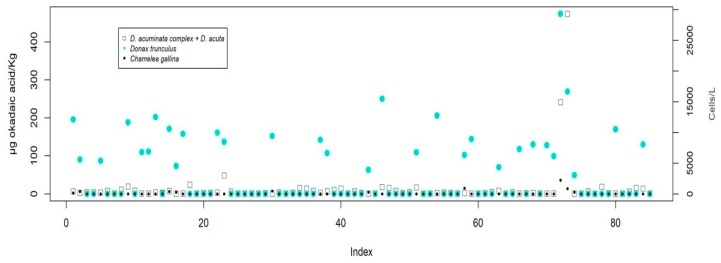
Toxicity in *D. trunculus* and *C. gallina* in simultaneous samples taken in the same area (x-axis: Sampling identification number). Accompanying the results is the concentration of *D. acuminata* complex being the majority *Dinophysis* species in the water sample taken at the same time.

The time series of contamination episodes of bivalve molluscs, together with the abundance of DSP-producing microalgae is shown in Appendix A. It shows how smaller bivalve species (larger specimens rarely exceed 3–4 cm in their axis of maximum growth) are more prone to the accumulation of lipophilic toxins. In this way, maximum levels of toxicity (µg okadaic acid/kg) were 2665 in *D. trunculus*, 913 in *C. gallina* and 1468 in *C. edule*, whereas in larger species, there were 687 in *M. galloprovincialis*, 91 in *C. chione* and 28 in *V. verrucosa*. This difference has been verified for the pair *D. trunculus* and *C. gallina* (Figure 5) in 85 double samples taken, which has contributed to the selection of *Donax trunculus* as an indicator species for some harvesting areas.

**Figure 6:**
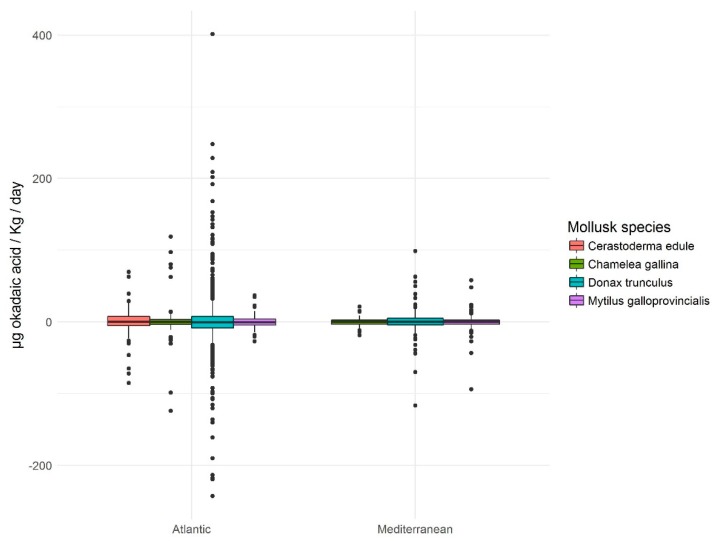
Box and whiskers of daily rate of accumulation / elimination of toxins (okadaic acid). In the same way that in Figure 4 the mollusc species with the highest toxic incidence have been selected, discarding those with a low tendency to accumulate.

In view of the results of the frequency distribution of accumulation and elimination daily rates (Figure 6), it is worth noting the symmetry and positive kurtosis. This means that, on the one hand, there are no appreciable differences between the speed of accumulation and elimination and, on the other hand, the values that are statistically considered as normal, do not exceed 25 µg okadaic acid/kg/day which, as has been said, are low values taking into account the extent of the range. This means that the occasions on which, starting from an absence of toxicity, the molluscs analyzed have the capacity to exceed the legal limit (160 µg okadaic acid/kg) for less than a week are statistically not significant (outliers; Figure 6).

The monitoring program of Andalusia establishes that once levels above the regulatory limit are detected in molluscs, temporary closures of fisheries must be triggered. They have been recorded in digital databases from 2007. The data presented (Table 2 and Table 3) correspond to this period.

## 3. Discussion

Data from monitoring programs are usually complex in their processing. Regulatory changes lead to modifications in sampling plans in a way that they lose their regularity when frequency, spatial distribution or target species change. This study has taken into account such features when calculating parameters or obtaining general conclusions.

The polymorphic life cycle, feeding behaviour and phase of the cell cycle have been given by various authors as the causes of the wide morphological variation of certain species of *Dinophysis* [16,17]. This variation makes some species intermediate forms similar in shape, a fact that makes it often difficult to discriminate them as it is the case with the pairs: *D. acuminata*/*D. sacculus*, *D. acuminata*/*D. ovum*, *D. sacculus*/*D. pavilardii*, *D. caudata*/*D.tripos* mentioned by various authors according to the case [13,14,18,19]. For a monitoring program, this difficulty translates into the possibility of producing false positive or negative results in the risk assessment, taking into account possible differences in the toxic potential between species. An example of this was resolved in the Thermaikos Gulf, Greece [20] by considering *D. acuminata* and *D. ovum* as *D.* cf *acuminata*. A similar treatment has been decided in the monitoring program of Andalusia where *D. acuminata*, *D. ovum* and *D. sacculus* are treated as a whole as *D. acuminata* complex.

The species associated with DST outbreaks in Andalusia (*D. acuminata* complex and *D. acuta*) have also been considered as the main species responsible on the Galician coast in addition to *D. sacculus* [17,19,21]. Vale and Sampayo [22,23] consider *D. acuminata* and *D. acuta* as responsible for specific DST episodes along the Portuguese coast. These two species are considered again to be responsible for similar outbreaks in Sweden [24]. In the North Sea, around the island of Helgoland (Germany), *D. norvegica* joins *D. acuminata* as co-responsible for episodes of contamination in mussels. *D. acuminata* appears as the species of wider distribution concerning its association with toxic events. However, there are cases in which the responsibility of episodes of intoxication is attributed to less common species such as *D. fortii*, *D. rotundata*, *D. caudata*, *D. sacculus* and *D. tripos* in the east Adriatic [25]. In our program, although such species have been detected, there were two results: toxicity was not observed in molluscs or the above-mentioned species of *Dinophysis* accompanied *D. acuminata* and/or *D. acuta* to a much lesser abundance.

It is important to mention the spatial coherence of the homogeneous areas obtained in the cluster analysis as there is no cluster covering non-neighbouring stations. It is also noteworthy that clusters mainly occupy coastal areas where the direction of the coast line is constant and that changes in that direction generate a different cluster. This feature seems to define a characteristic length of cluster dependent on such geomorphological characteristics that, in turn, could define hydrodynamic characteristics. This gives consistency to the conclusion derived from the analysis that the patterns and abundances of *D. acuminata* complex in the different stations of each cluster are similar.

The seasonal patterns of *Dinophysis* (growth pulses from the beginning of the spring until the end of autumn) in Andalusia do not differ from those found by the other authors in western Europe. It exceeds at the end points of the period mentioned by Smayda [26]: “the blooms of dinoflagellates occur in predominantly warm waters which are stratified from late spring to early autumn”. Other authors have limited this same period as the one with the largest development of *Dinophysis* [27,28,29,30,31]. In general, although the most apparent growth peaks are given within this period, species such as *D. acuminata* complex are present throughout the rest of the year even though at very low concentrations. Nincevic-Gladan [25] revealed that of the 13 species detected in the east Adriatic coast, only *D. tripos* appeared in winter. Koukaras [20] noted that, at genus level, the episodes usually lasted between 3 and 4 months, and sporadically appeared throughout the rest of the year.

Many authors have reported and analysed the possible causes of the discrepancy between the values for toxicity in molluscs and the abundance of microalgae [24,32,33,34]. The causes mainly proposed are the presence of alternative food, environmental factors, the stratification of the water column that favours the development of thin layers of microalgae, or the variability in the toxin content per cell. In addition, other authors have studied the daily vertical migration of plankton [13,35,36,37,38], which translates into irregularity in daily food availability for molluscs. Discrepancy in the pairs of parallel sampling values (if a linear correlation is expected between cell numbers and toxicity) and the explanation of the inaccuracy of dinoflagellate abundance as an indicator of the true exposure suffered by molluscs throughout the previous week has been found in our data. This discrepancy may be a consequence of the factors above mentioned. In this way, the toxicity value in molluscs represents the result of a continuous exposure before the sample is taken, while the value of plankton concentration is representative of the moment of capture as molluscs have not yet responded.

The European regulations [3,4] says “production areas must be periodically monitored to check for the presence of toxin-producing plankton in production and relaying waters and biotoxins in live bivalve molluscs. The sampling frequency for toxin analysis in the molluscs is, as a general rule, to be weekly during the periods at which harvesting is allowed. This frequency may be reduced in specific areas, or for specific types of molluscs, if a risk assessment on toxins or phytoplankton occurrence suggests a very low risk of toxic episodes”. On the other hand, it indicates that monitoring must be increased if there is evidence or suspicions of an increased risk. Such indications, in principle, leave no room for a lack of efficiency in the programs established at European level so, the answer to the initial question raised in this study, “Are the regulatory requirements for the analysis of toxic phytoplankton effective as an early-warning system of the risk of contamination of molluscs in the Andalusian coast?” would be “Yes”. The problem lies in the risk assessment. In Andalusia, the check once a week of the concentration of phytoplankton, as discussed above, may have inaccuracies as indicators. An important number of cases in which molluscs had the capacity to exceed the legal limits in less than a week have been detected on our coast. Under these conditions, 11.6% of the cases were of *Donax trunculus*, 12.2% of *Cerastoderma edule*, 11.5% of *Mytilus galloprovincialis* and 11.4% of *Chamelea gallina*. Therefore, in order to prevent, to some extent, this risk from 2015 on, the uncertainty of the measure of toxicity needed to activate the intensification of sampling (48 h) was set in the criteria of the monitoring program as a factor to take into account. On the other hand, incidents with the toxicities of bivalve molluscs have been so important that there have been closures of fisheries for even half a year. A fact that shows the magnitude of the problem, bearing in mind that such fisheries represent a significant economic source at a local coastal scale. As has been mentioned, there have been numerous studies aimed at understanding the dynamics of the plankton’s and molluscs’ toxicities. Although there is increasing awareness of them, it remains necessary to expand knowledge which, with large sets of data of different nature, will shed light to the production of other indices or will suggest changes in sampling strategies to reduce the range of health risk. 

## 4. Materials and Methods 

All the data that underpin this article correspond to the information generated by the program for phytoplankton and biotoxins official monitoring established in Andalusia in 1994. The data generated were not recorded on a computer system until 1999. Therefore, this article refers to the period from then on. The information is available to the public and can be found at the web page http://www.cap.junta-andalucia.es/agriculturaypesca/moluzonasprodu/ of the Regional Ministry for Agriculture, Fisheries and Rural Development of Andalusia (Regional Government). That Ministry is conferred with the exclusive jurisdiction for fishing in inland waters, shellfish and aquaculture. The laboratory designated by the competent authority to perform the analyses corresponding to the official monitoring program is the *Laboratory for the Quality Control of Fishery Resources* which has developed its functions since 1996. The laboratory has been entitled to develop this monitoring since 2007, according to the UNE-EN-ISO 17025 standard.

### 4.1. Sampling Plan

**Figure 7:**
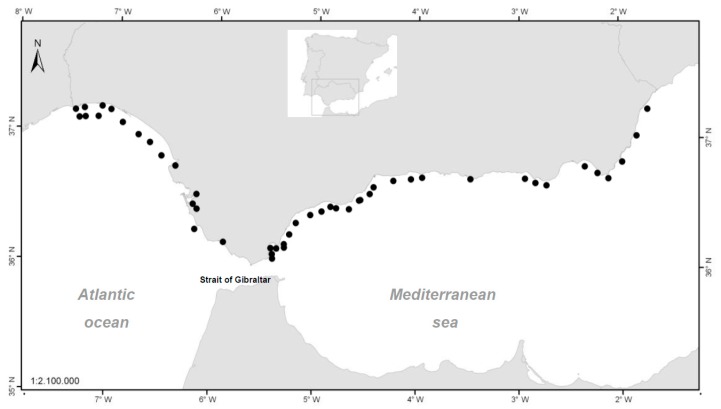
Centroids of the production areas declared by the Order of 15 July 1993 - BOJA (Spanish acronym for the Andalusian Official Gazette) No. 85 of 5 August 1993 [39]—which have been updated in successive orders.

The sampling stations in Figure 7 are represented by the centroid of the production area to which they belong. However, the point of capture of each sample can be placed in any position within the same area.

Based on the operational capacity of the program, the sampling strategy has been adapted as far as possible to the European Regulations and their successive modifications. Thus, the sampling frequency was weekly at some stations from the beginning while others started on a fortnightly basis for water and quarterly for molluscs. However, this frequency has now been increased to include sampling all parameters in all production areas declared every week. The program has always allowed the possibility of increasing sampling if evidence of an increase of a toxicological risk is detected. The successive changes are detailed in Appendix C (Table A1, Table A2, Table A3 and Table A4).

### 4.2. Sampling Techniques

Water samples were collected using vertical hauls with bongo nets of mesh size 20 μm and a hose system [40]. Samples of molluscs from natural populations were collected using techniques traditionally used by the Andalusian shellfish sector. Samples were either taken at points of highest productive activity within each production area or provided by producers from their aquaculture facilities.

### 4.3. Analysis

Testing of lipophilic toxins and toxic phytoplankton are based on official methods harmonized and accredited since 2007 by ENAC (National Accreditation Entity-in Spanish) according to the standard UNE-EN-ISO 17025.

For the identification and enumeration of *Dinophysis* spp., the technique of Utermöhl [41] was used as recommended in European standard UNE-EN 15204 *Guidance standard on the enumeration of phytoplankton using inverted microscopy*.

Lipophilic toxin levels were determined according to the method described by Yasumoto et al. [42]. From 2011 on, the data presented correspond to analyses carried out using the European harmonized chemical technique (LC-MS/MS).

### 4.4. Data Processing

The processing and graphic presentations of the data are carried out using the free software for statistical analysis and graphics R in its 2013 version [15].

Given the huge volume of records (20 years), and for the submission of the time series, a cluster analysis was carried out on producing areas in order to reduce the datasets to be presented, excluding redundancy without losing relevant information. To do this, the K-means non-hierarchical cluster analysis (function in R: kmeans()) was used after developing the analysis of the optimal number of clusters (function in R: cascadeKM()) over a maximum of 12 clusters. (If more clusters were extracted, the purpose sought with the use of this technique would not be achieved). This is an unsupervised automatic method for pattern recognition, i.e., it is based on the response variable (concentration of *D. acuminata* complex) without seeking correlations with explanatory variables (e.g., environmental). For the response variable, the concentration of *Dinophysis acuminata* complex was chosen, as it provides more information because it has a high frequency in the samples. A total of 6659 samples were used after excluding very low incidence areas (see Figure 3) and extracting only quantifiable values. Thus, it is assumed that the homogeneous areas defined by *D. acuminata*, probably conditioned by the hydrodynamic characteristics, could be homogeneous also for the other species. In any case, the most relevant species in Andalusia is *D. acuminata* complex and the presentation of the information must adapt mainly to it. To obtain the optimal number of areas the “SSI” (Simple Structure Index) criterion has been used [43]. This is an index that combines three factors: 1) the greatest difference in the response variable between clusters, 2) the sizes of the most contrasting clusters and 3) the deviation of the variable in the cluster centers compared to its overall mean. It can be deduced that each factor separately indicates a more homogeneous selection of groups and thus, a higher SSI score implies a better partition.

The calculation of the daily rate of accumulation or elimination of toxins is carried out through the difference in toxicity measured between the samples from the same area in two successive samplings divided by the number of days, provided that between the two sampling there are not more than 11 days (maximum amplitude in samples obtained in working days of two consecutive weeks). This rate is a calculated value that indicates what would be the accumulation or elimination if every day between the two samples was kept constant. It represents, therefore, a theoretical daily value that must be taken with caution, understanding that there are probably days between the two samples with a higher and lower rate.

The information provided concerning the relative sensitivity among bivalve species identically exposed to microalgae of *Dinophysis* genus derives from parallel samples, if present, in which such species have been collected at the same time in the same area.

## Figures and Tables

**Figure 2:**
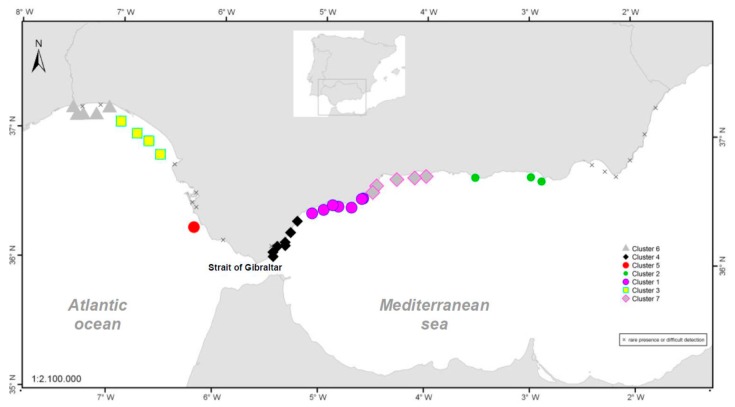
Result of the cluster analysis using the k-means non-hierarchical method. The areas excluded from the analysis (marqued with an “x”) are those in which the presence of *Dinophysis* was detected with a very low frequency for various reasons: impossibility of quantifying samples due to organic matter or areas of low sampling frequency due to the absence of extractive activity.

**Table 1:** Species of the genus *Dinophysis* found in the samples. (34,329 samples).

Species	Frequency in Atlantic (%)	Frequency in Mediterranean (%)
*Dinophysis acuminata* Claparède & Lachmann, 1859	85.2	53.8
*Dinophysis acuta* Ehrenberg, 1839	19.4	9.6
*Dinophysis argus (Phalacroma argus)* F. Stein 1883	0.1	0.9
*Dinophysis caudata* Saville-Kent, 1881	54.7	55.3
*Dinophysis amphora* Balech 1971	<0.1	0
*Dinophysis schroederi* J. Pavillard 1909	0.2	0.9
*Dinophysis cuneus (Phalacroma cuneus)* F. Schütt 1895	0	0.1
*Dinophysis doryphora (Phalacroma doryphorum)* Stein 1883	0.2	0.9
*Dinophysis fortii* Pavillard, 1923	4.5	17.1
*Dinophysis hastata* F. Stein 1883	0.8	0.3
*Dinophysis odiosa* (Pavillard) Tai & Skogsberg 1934	3.3	0.5
*Dinophysis pusilla* Jörgensen 1923	<0.1	0.2
*Dinophysis rapa (Phalacroma rapa)* F. Stein 1883	0.2	6.8
*Dinophysis sacculus* Stein, 1883	2.5	2.7
*Dinophysis schuettii* G. Murray & Whitting 1899	4.7	5.6
*Dinophysis similis* Kofoid & Skogsberg 1928	<0.1	0.1
*Dinophysis tripos* Gourret, 1883	<0.1	2.1
*Dinophysis mitra (Phalacroma mitra)* F. Schütt, 1895	0.1	0.7
*Phalacroma rotundatum (Dinophysis rotundata)* (Claparéde & Lachmann) Kofoid & Michener, 1911	12.5	5.1
*Dinophysis ovum*^1^ (F. Schütt) T.H. Abé	(^1^)	(^1^)
*Dinophysis* spp.	42.2	46.2

^1^ Species identified from isolated specimens and kept in culture. Routinely, *Dinophysis ovum* are included in the *Dinophysis acuminata* complex.

**Table 2:** Maximum annual closing period (days) of a fishery in an Atlantic area from 2007 to 2018.

Species ^1^	2007	2008	2009	2010	2011	2012	2013	2014	2015	2016	2017	2018
*Donax trunculus*	161	63	51	99	45	112	55	126	125	47	46	-
*Chamelea gallina*	196	194	40	34	85	63	22	126	41	47	73	-
*Mytilus galloprovincialis*	0	0	0	54	13	147	63	4	27	34	0	-
Cerastoderma edule	0	0	0	0	34	14	34	43	30	32	0	0

^1^ The mollusc species with the highest toxic incidence are shown, discarding those with a low tendency to accumulate. The latter are: *Ruditapes philippinarum, Scrobicularia plana, Polititapes rhomboides, Ruditapes decussatus, Dosinia* spp, *Polititapes aureus, Magallana gigas, Mimachlamys varia, Solen marginatus, Venus verrucosa, Acanthocardia tuberculata, Aequipecten opercularis and Pecten maximus.*

**Table 3:** Maximum annual closing period (days) of a fishery in a Mediterranean area from 2007 to 2018.

Species ^1^	2007	2008	2009	2010	2011	2012	2013	2014	2015	2016	2017	2018
*Donax trunculus*	0	0	18	0	119	48	90	77	11	34	14	0
*Chamelea gallina*	0	0	18	0	126	51	113	77	71	34	14	0
*Mytilus galloprovincialis*	0	0	0	10	64	59	50	55	0	16	3	0
Callista chione	0	0	95	0	171	92	85	77	11	77	14	0
Venus verrucosa	0	0	0	0	98	32	113	59	0	34	14	0

^1^*The same as in Table 2*.

**Figure A1:**
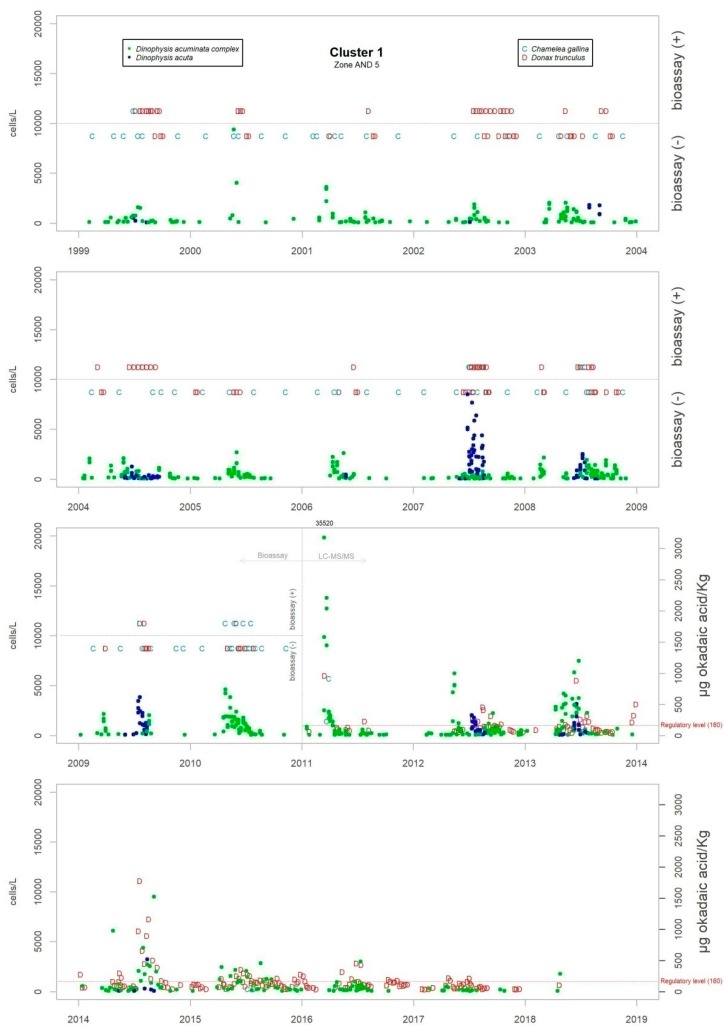
Temporal series of the abundance of *D. acuminata* and *D. acuta* and the concentration of okadaic acid in the species of mollusc monitored in each harvest area, in cluster 1. The order of the sequence of figures from Figure A1 to Figure A7 follows the geographic order of the clusters from west to east.

**Figure A2:**
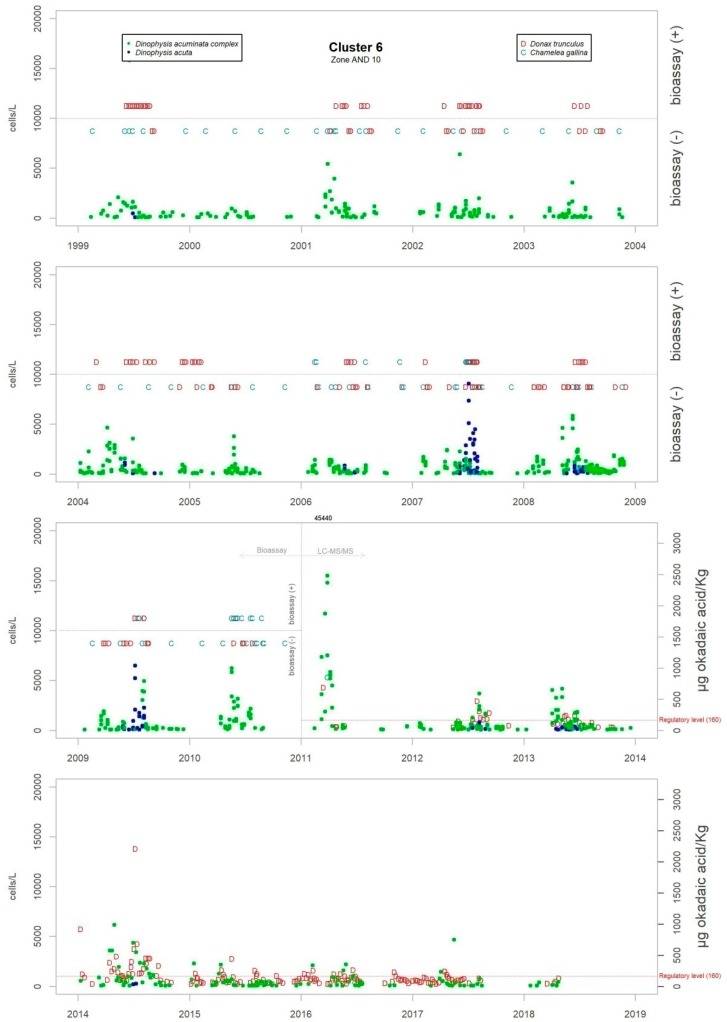
Temporal series of the abundance of *D. acuminata* and *D. acuta* and the concentration of okadaic acid in the species of mollusc monitored in each harvest area, in cluster 6. The order of the sequence of figures from Figure A1 to Figure A7 follows the geographic order of the clusters from west to east.

**Figure A3:**
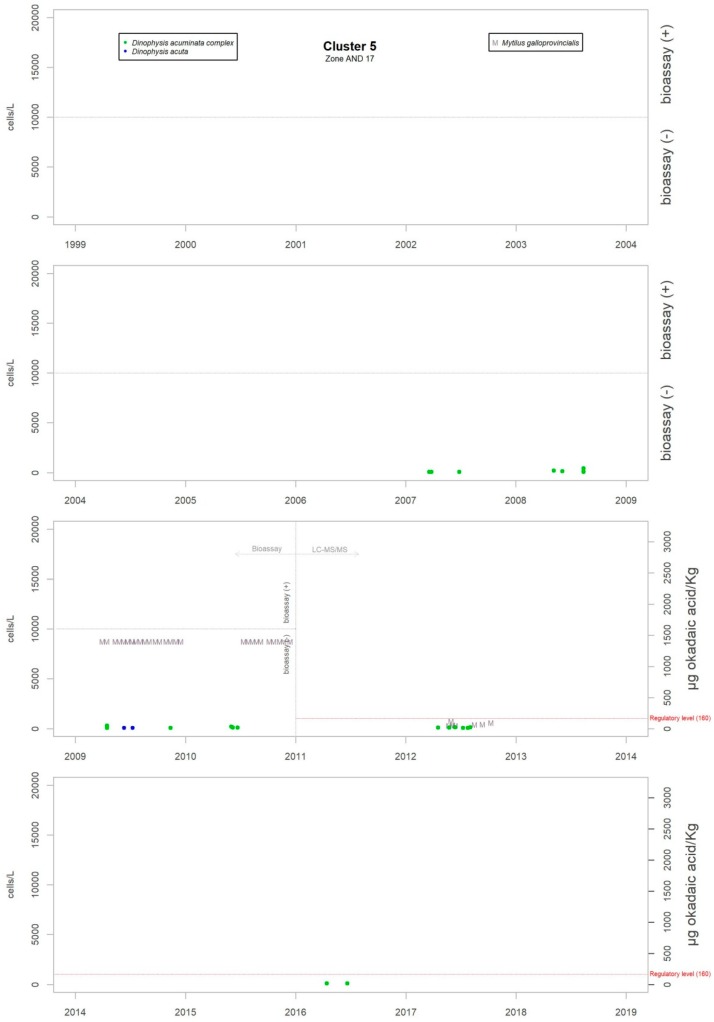
Temporal series of the abundance of *D. acuminata* and *D. acuta* and the concentration of okadaic acid in the species of mollusc monitored in each harvest area, in cluster 5. The order of the sequence of figures from Figure A1 to Figure A7 follows the geographic order of the clusters from west to east. The long periods of absence correspond to lack of sampling due to cessation of harvesting.

**Figure A4:**
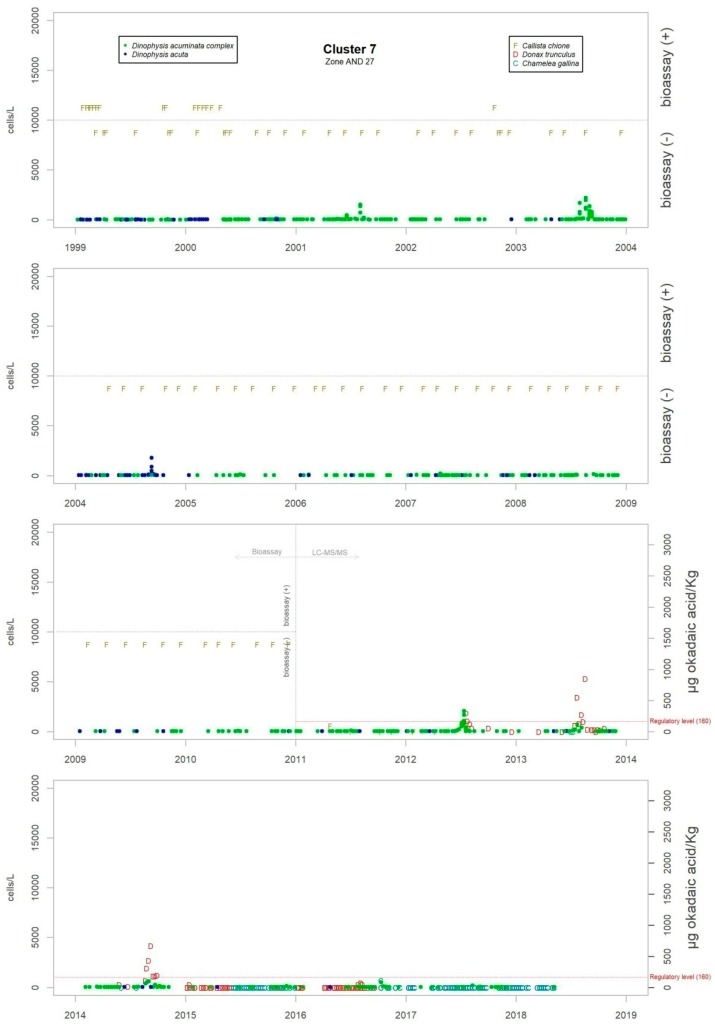
Temporal series of the abundance of *D. acuminata* and *D. acuta* and the concentration of okadaic acid in the species of mollusc monitored in each harvest area, in cluster 7. The order of the sequence of figures from Figure A1 to Figure A7 follows the geographic order of the clusters from west to east.

**Figure A5:**
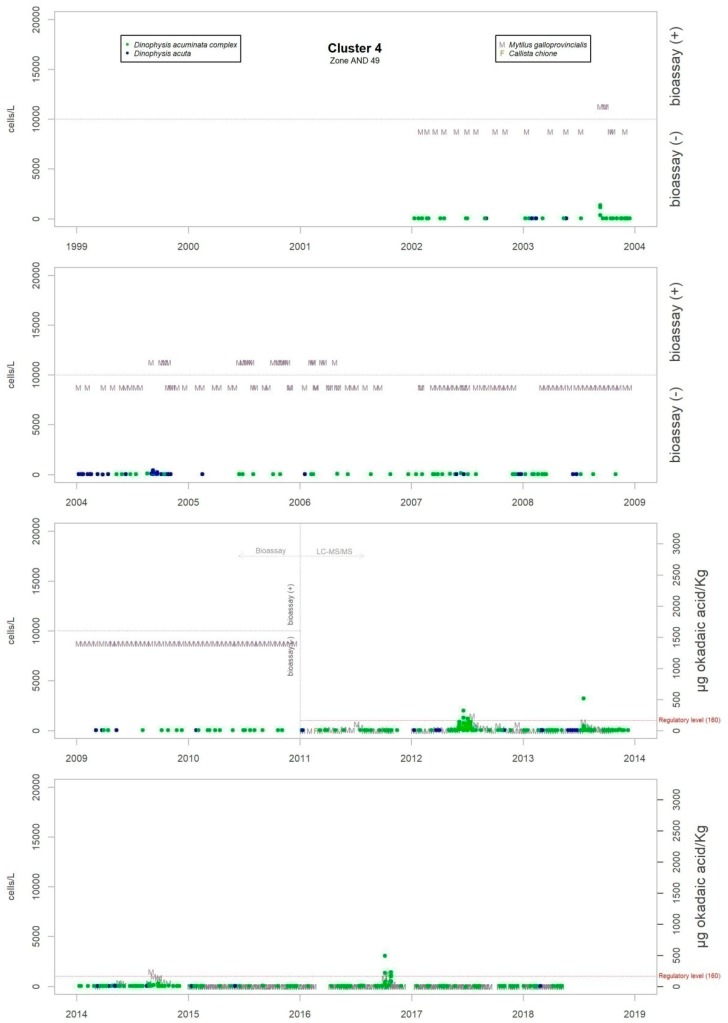
Temporal series of the abundance of *D. acuminata* and *D. acuta* and the concentration of okadaic acid in the species of mollusc monitored in each harvest area, in cluster 4. The order of the sequence of figures from Figure A1 to Figure A7 follows the geographic order of the clusters from west to east.

**Figure A6:**
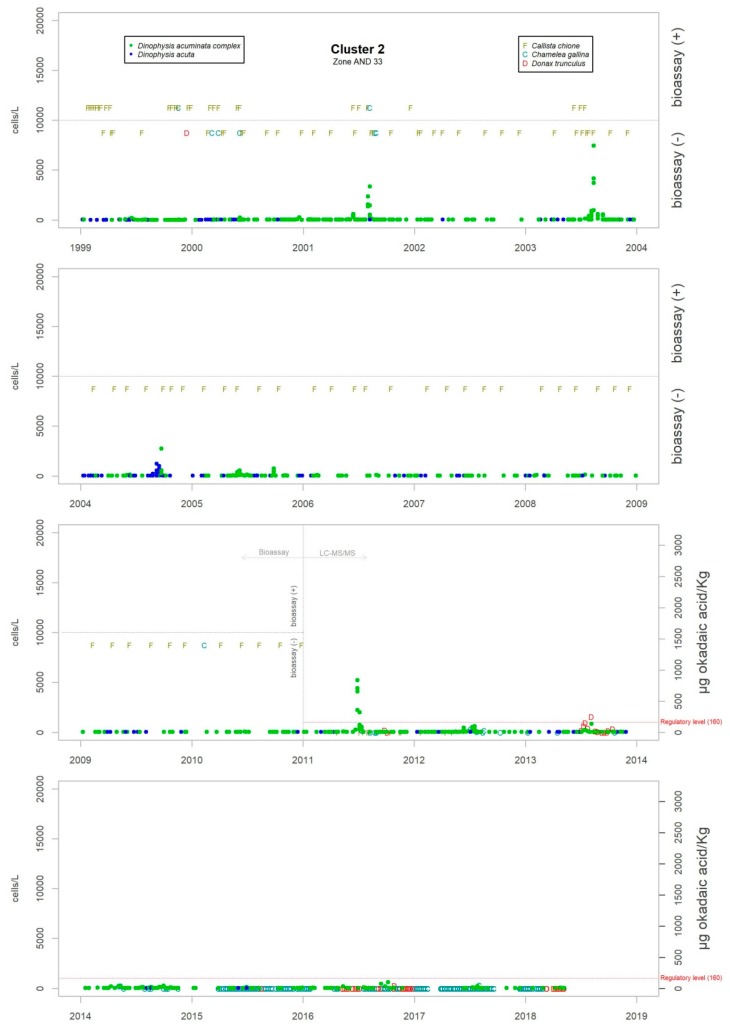
Temporal series of the abundance of *D. acuminata* and *D. acuta* and the concentration of okadaic acid in the species of mollusc monitored in each harvest area, in cluster 2. The order of the sequence of figures from Figure A1 to Figure A7 follows the geographic order of the clusters from west to east.

**Figure A7:**
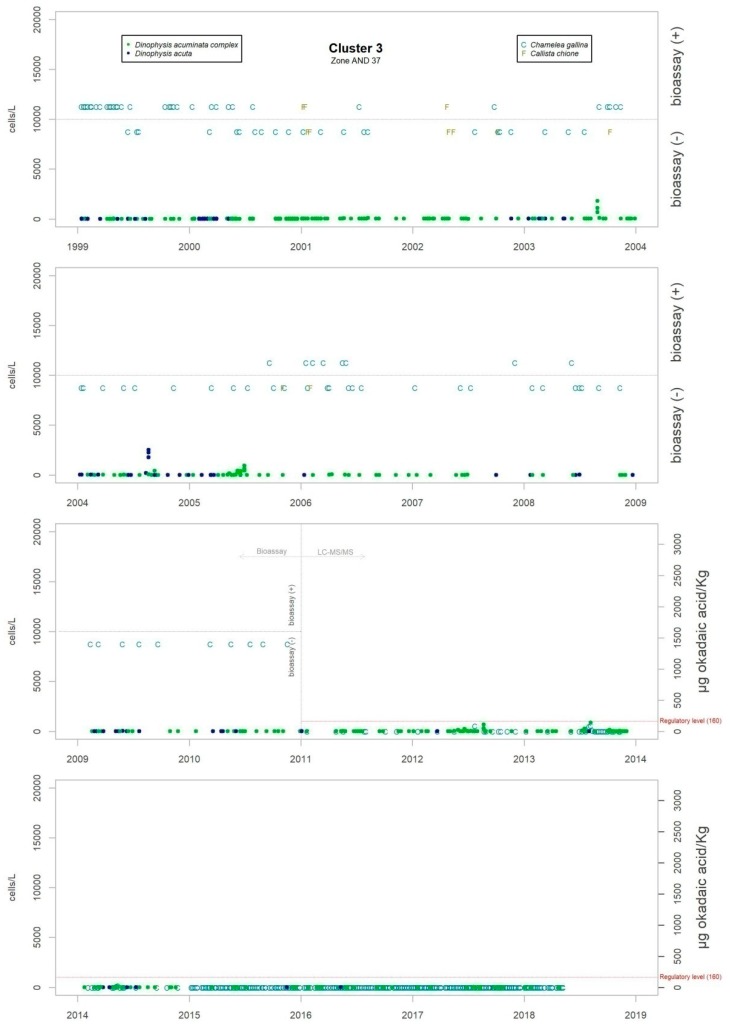
Temporal series of the abundance of *D. acuminata* and *D. acuta* and the concentration of okadaic acid in the species of mollusc monitored in each harvest area, in cluster 3. The order of the sequence of figures from Figure A1 to Figure A7 follows the geographic order of the clusters from west to east.

**Figure A8:**
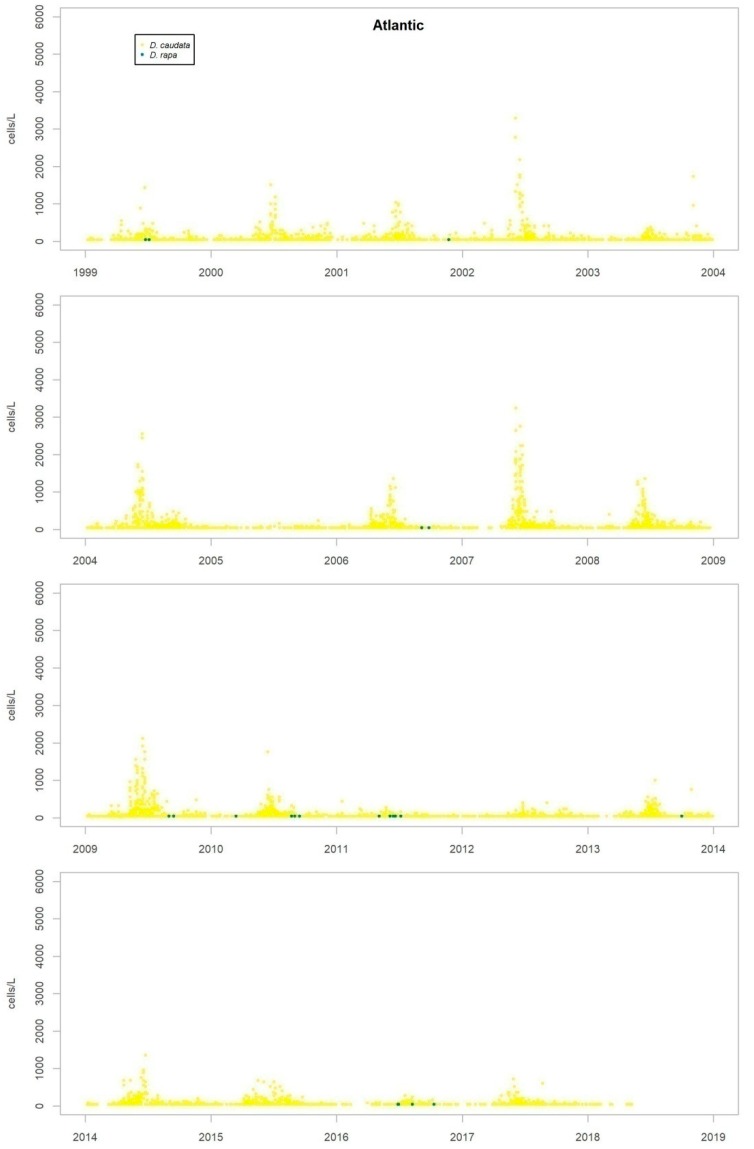
Temporal series of the abundance of *D. caudata* and *D. rapa* in the areas of the Atlantic Ocean.

**Figure A9:**
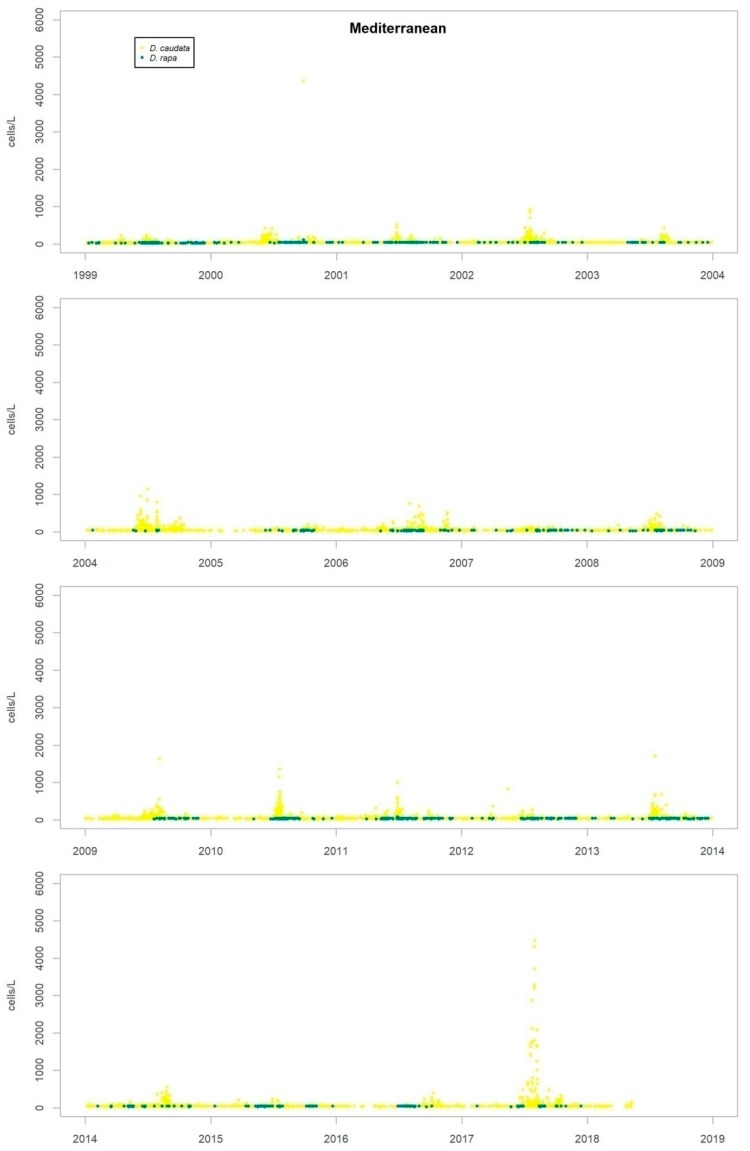
Temporal series of the abundance of *D. caudata* and *D. rapa* in the areas of the Mediterranean Sea.

**Figure A10:**
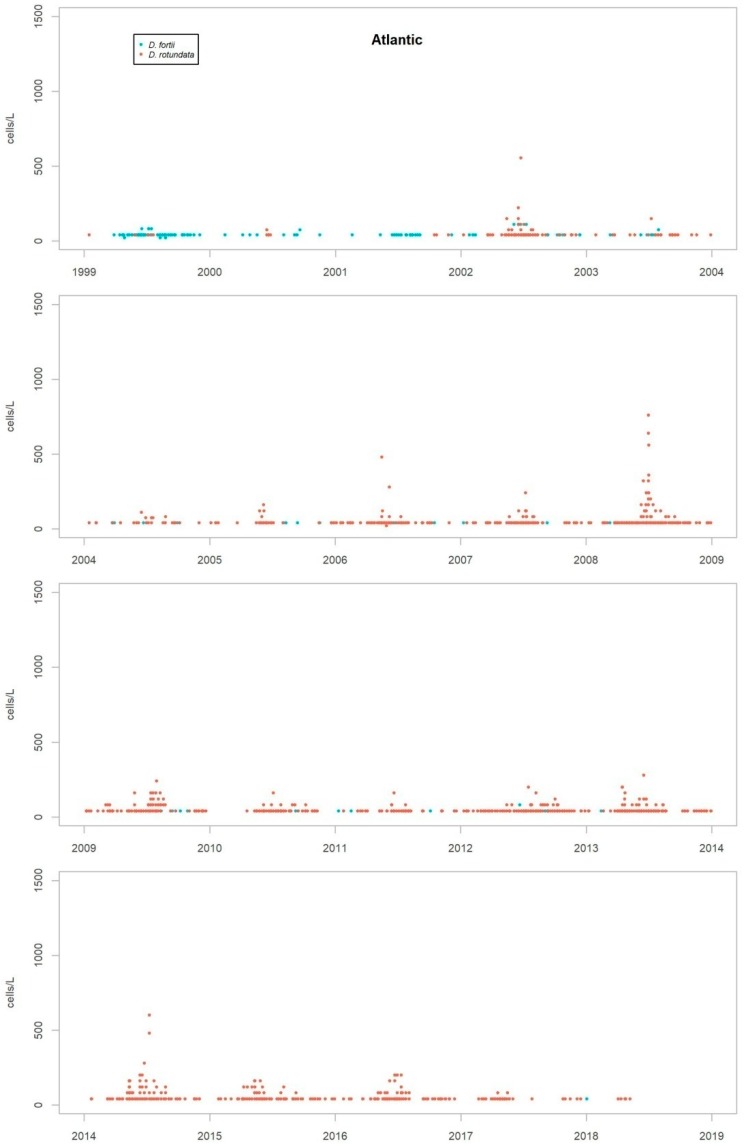
Temporal series of the abundance of *D. fortii and D. rotundata* in the areas of the Atlantic Ocean.

**Figure A11:**
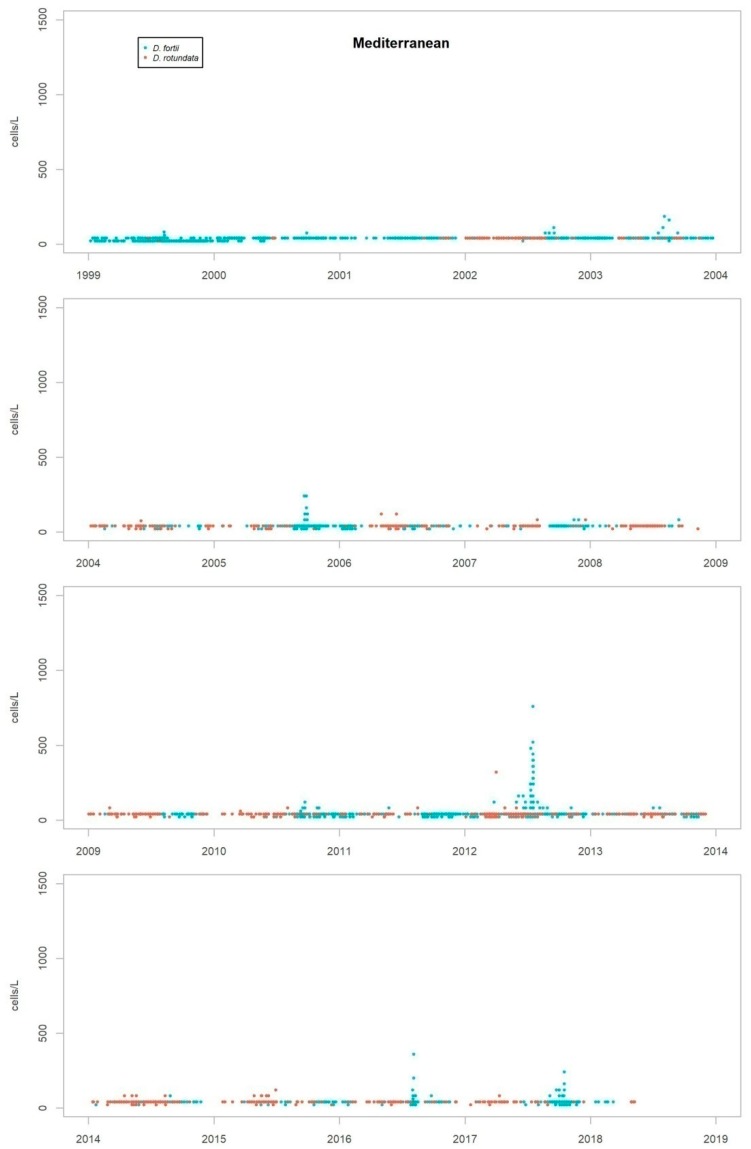
Temporal series of the abundance of *D. fortii and D. rotundata* in the areas of the Mediterranean Sea.

## Appendix C

**Table A1:** Sampling frequencies in marine waters for the phytoplankton analysis from 1999 to 2008.

Frequency	1999	2000	2001	2002	2003	2004	2005	2006	2007	2008
Weekly	1–13, 21–24, 27–36 ^1^	1–13, 21–24, 27–36	1–13, 21–24, 27–36	1–13, 21–24, 27–36	1–13, 21–24, 27–36	1–13, 21–24, 27–36	1–13, 21–24, 27–38, 40, 44	1–13, 21–24, 27–38, 40, 44	1–13, 21–24, 27–38, 40, 44	1–13, 21–24, 27–38, 40, 44
Biweekly	37–44	37–44	37–44	37–44	37–44	37–44	14–16, 18, 39, 41–43	14–16, 18, 39, 41–43	14–16, 18, 39, 41–43	14–16, 18, 39, 41–43
Monthly	14–20	14–20	14–20	14–20	14–20	14–20	17, 19, 20	17, 19, 20	17, 19, 20	17, 19, 20

^1^ Data on the cells, identification number of the harvest area. To avoid clutter, the prefix AND- (through which they are officially known) has been omitted.

**Table A2:** Sampling frequencies in marine waters for phytoplankton analysis from 2009 to 2018.

Frequency	2009	2010	2011	2012	2013	2014	2015	2016	2017	2018
Weekly	1–13, 21–24, 27–38, 40, 44 ^1^	1–13, 21–24, 27–38, 40, 44	1–13, 21–24, 27–38, 40, 44	1–13, 21–24, 27–38, 40, 44	All active zones	All active zones	All active zones	All active zones	All active zones	All active zones
Biweekly	14–16, 18, 39, 41–43	14–16, 18, 39, 41–43	14–16, 18, 39, 41–43	14–16, 18, 39, 41–43						
Monthly	17, 19, 20	17, 19, 20	17, 19, 20	17, 19, 20						

^1^ Data on the cells, identification number of the harvest area. To avoid clutter, the prefix AND- (through which they are officially known) has been omitted.

**Table A3:** Sampling frequencies of molluscs for the analysis of lipophilic toxins from 1999 to 2008.

Frequency	1999	2000	2001	2002	2003	2004	2005	2006	2007	2008
Weekly										
Monthly	36 ^1^	36	36	36	36	36	4, 7, 11, 24, 30	4, 7, 11, 24, 30	4, 7, 11, 24, 30	4, 7, 11, 24, 30
Bimonthly							24, 27–35, 37–44	24, 27–35, 37–44	24, 27–35, 37–44	24, 27–35, 37–44
Quarterly	1–35, 37–44	1–35, 37–44	1–35, 37–44	1–35, 37–44	1–35, 37–44	1–35, 37–44	1–3, 5, 6–10, 13–22	1–3, 5, 6–10, 13–22	1–3, 5, 6–10, 13–22	1–3, 5, 6–10, 13–22

^1^ Data on the cells, identification number of the harvest area. To avoid clutter, the prefix AND- (through which they are officially known) has been omitted.

**Table A4:** Sampling frequencies of molluscs for the analysis of lipophilic toxins from 2009 to 2018.

Frequency	2009	2010	2011	2012	2013	2014	2015	2016	2017	2018
Weekly					All active zones ^1^	All active zones ^1^	All active zones ^1^	All active zones ^1^	All active zones ^1^	All active zones ^1^
Monthly	4, 7, 11, 24, 30	4, 7, 11, 24, 30	4, 7, 11, 24, 30	4, 7, 11, 24, 30						
Bimonthly	24, 27–35, 37–44	24, 27–35, 37–44	24, 27–35, 37–44	24, 27–35, 37–44						
Quarterly	1–3, 5, 6–10, 13–22	1–3, 5, 6–10, 13–22	1–3, 5, 6–10, 13–22	1–3, 5, 6–10, 13–22						

^1^ Data on the cells, identification number of the harvest area. To avoid clutter, the prefix AND- (through which they are officially known) has been omitted. In areas of bivalve molluscs from aquaculture, monitoring was activated only during the production season.
